# Antigen Cross-Presentation of Immune Complexes

**DOI:** 10.3389/fimmu.2014.00140

**Published:** 2014-04-01

**Authors:** Barbara Platzer, Madeleine Stout, Edda Fiebiger

**Affiliations:** ^1^Department of Pediatrics, Division of Gastroenterology and Nutrition, Boston Children’s Hospital, Harvard Medical School, Boston, MA, USA

**Keywords:** anti-tumor immune responses, DC subset functions, cell type-specific cross-presentation, IgG-complexed antigens, Fc receptor-mediated antigen uptake, CD8^+^ T cell priming

## Abstract

The ability of dendritic cells (DCs) to cross-present tumor antigens has long been a focus of interest to physicians, as well as basic scientists, that aim to establish efficient cell-based cancer immune therapy. A prerequisite for exploiting this pathway for therapeutic purposes is a better understanding of the mechanisms that underlie the induction of tumor-specific cytotoxic T-lymphocyte (CTL) responses when initiated by DCs via cross-presentation. The ability of humans DC to perform cross-presentation is of utmost interest, as this cell type is a main target for cell-based immunotherapy in humans. The outcome of a cross-presentation event is guided by the nature of the antigen, the form of antigen uptake, and the subpopulation of DCs that performs presentation. Generally, CD8α^+^ DCs are considered to be the most potent cross-presenting DCs. This paradigm, however, only applies to soluble antigens. During adaptive immune responses, immune complexes form when antibodies interact with their specific epitopes on soluble antigens. Immunoglobulin G (IgG) immune complexes target Fc-gamma receptors on DCs to shuttle exogenous antigens efficiently into the cross-presentation pathway. This receptor-mediated cross-presentation pathway is a well-described route for the induction of strong CD8^+^ T cell responses. IgG-mediated cross-presentation is intriguing because it permits the CD8^−^ DCs, which are commonly considered to be weak cross-presenters, to efficiently cross-present. Engaging multiple DC subtypes for cross-presentation might be a superior strategy to boost CTL responses *in vivo*. We here summarize our current understanding of how DCs use IgG-complexed antigens for the efficient induction of CTL responses. Because of its importance for human cell therapy, we also review the recent advances in the characterization of cross-presentation properties of human DC subsets.

## Introduction

The mechanism of cross-presentation allows exogenous antigens to access the processing and presentation machinery of a cell so that exogenous antigenic peptides are displayed on MHC class I molecules for T cell recognition, which consequently leads to the priming of CD8^+^ T cell responses. As such, the cross-presentation pathway is essential for inducing cytotoxic T-lymphocyte (CTL) responses against viruses as well as intracellular bacteria, which do not infect the APC (1–4). Additionally, cross-presentation is thought to be crucial in mounting immune responses against tumor antigens. Indeed, cross-priming of tumor reactive cytotoxic CD8^+^ T cells through cell-based tumor vaccines is a major goal in cancer immunotherapy (5, 6). Induction, the so called priming, of tumor-specific CD8^+^ T cells is an appealing therapeutic strategy because the generated CTLs not only mediate antigen-specific killing of the targeted tumor via cell–cell contacts, but also provide the host with long-lasting memory responses that may prevent cancer recurrence.

Dendritic cells (DCs) have been proven to be superior in routing exogenous protein antigen toward cross-presentation; however, they comprise a heterogeneous cell population, and significant differences in the cross-presentation capacity of different DC subsets have been reported (4). Importantly, cross-presentation of antigen does not result solely in the priming of CTLs but can also lead to the induction of cross-tolerance (7). The latter immunological outcome should by all means be avoided during cancer therapy. Thus, to take full advantage of the therapeutic potential of antigen cross-presentation by DCs, significant effort was made to delineate precisely how cross-presentation is initiated and regulated. By now, many mechanistic details of antigen cross-presentation have been discovered whereas others still remain enigmatic. In contrast to MHC class II-restricted antigen presentation, the default pathway for the display of exogenous antigens for immune recognition and the induction of CD4^+^ T cell responses, cross-presentation *in vivo* is thought to be controlled rather strictly by the type of DCs used as antigen-presenting cells. In this review, we summarize the current knowledge on how immune complexes facilitate antigen cross-presentation and expand the cross-presentation capacity of specific DC subsets. We also discuss the therapeutic potential of this cross-presentation pathway.

## IgG Immune-Complexed Antigens Enter the Cross-Presentation Pathway through Fc Receptors

**Figure 1:**
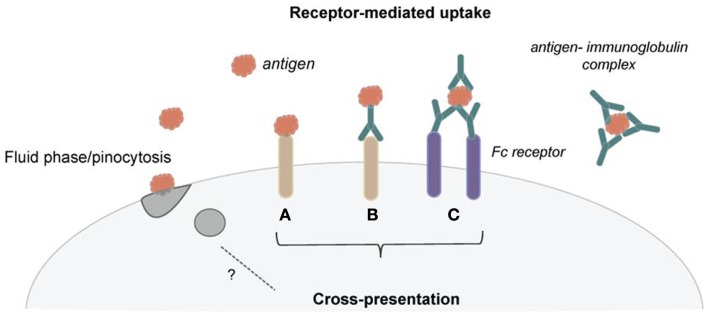
**Dendritic cells use several mechanisms of antigen uptake for cross-presentation**. **(A)** Several receptors have been shown to efficiently shuttle exogenous antigen into the cross-presentation pathway. **(B)** These receptors are now employed to target DCs *in vivo* for cancer immunotherapy using receptor-specific antibodies coupled with antigen. **(C)** Immunoglobulins can bind to antigen and form immune complexes. These immune complexes can then be taken up via Fc receptors and deliver antigen for cross-presentation. Pinocytosis seems not to be an effective mechanism for routing antigen toward cross-presentation.

Our immune system has to respond to a variety of different forms of antigens and thus has developed an array of mechanisms to deal with antigenic diversity. Antigens can be small soluble molecules, which are taken up by fluid phase mechanisms, or larger particles, such as bacteria, which are phagocytosed. To facilitate antigen uptake and processing, DCs also use an assortment of endocytic receptors (Figure 1). Several of these endocytic receptors belong to the C-type lectin family. For example, DEC-205, the mannose receptor, and Clec9a have been shown to efficiently shuttle antigen for cross-presentation. Several recent reviews give detailed insight into the functional differences of these endocytic receptors, and they are therefore only briefly mentioned here (8–10). Importantly, monoclonal antibodies against these endocytic receptors have been employed to target antigen to DCs for cross-presentation, and using this strategy, encouraging anti-tumor immunity was initiated in mice (11–13). Thus, strong emphasis is continuously put on targeting of cross-presenting DCs to elicit anti-tumor responses, as exhibited in several ongoing clinical trials (11, 14–16). A so far therapeutically less exploited but remarkably effective way for DCs to internalize antigen for cross-presentation is via Fc receptors (Figure 1). Antigens, especially under inflammatory conditions, can be found already bound to antigen-specific antibodies, and these antigen–antibody complexes (referred to as immune complexes or immune-complexed antigen) can be recognized by Fc receptors through the Fc region of the antibodies. Binding of the immune complexes typically triggers crosslinking of the Fc receptors, their internalization together with the antigen, and shuttling of the immune complexes toward antigen presentation compartments (17, 18).

Before the crucial role of Fc receptors in antigen cross-presentation was identified, their value in enhancing antibody-dependent cellular cytotoxicity (ADCC) by inflammatory cells, including neutrophils and macrophages, was already recognized (19). Enhancement of T cell proliferation via antigen-specific antibodies that bind Fc receptors became evident in the mid-1980s (20–22). Studies using Fcγ receptor knockout mice revealed the general requirement of Fcγ receptor engagement for the effectiveness of anti-tumor immune responses *in vivo*. The finding that anti-tumor antibodies require the induction of CTL responses to be effective suggested early on that Fcγ receptors contribute to anti-tumor immunity in addition to mediating ADCC (23). Shortly after, it was compellingly demonstrated that endocytosis of immune complexes via Fcγ receptor allows MHC class I-restricted antigen presentation and the priming of CTLs (24, 25). The finding that DCs use immunoglobulin G (IgG)-immune complexes to efficiently prime specific CD8^+^ CTL responses was shortly thereafter confirmed *in vivo* (26). Furthermore, it was shown that only antigen targeting to FcγR on DCs, but not antigen targeting to surface immunoglobulins on B cells, induces efficient cross-presentation, despite the fact that both targeting strategies allow these cell types to present antigen via MHC class II with equal efficiency (27).

The therapeutic potential of Fc receptor-mediated antigen uptake for anti-tumor immunotherapy became evident early on. Studies with human cells demonstrated that coating human myeloma cells with monoclonal antibodies promotes cross-presentation of myeloma-associated antigens by human DCs. The enhanced cross-presentation of tumor antigen was preventable by pretreatment of the DCs using Fcγ receptor blocking antibodies (28). Notably, this study did not observe that Fcγ receptor-mediated antigen uptake induces significant phenotypic maturation of human DCs, as it has been described for murine DCs (24, 26, 27). The possible absence of maturation induction in human DCs through immune complexes is important to keep in mind with regard to a clinical applicability of Fc receptor targeting. DC maturation in the context of antigen uptake is considered to be a crucial attribute that must be achieved to induce efficient CTL responses by cross-presentation receptors because otherwise cross-tolerance may be induced (7). Overall, although there is substantial evidence suggesting that cross-presentation of immune-complexed antigen via Fcγ receptors is a promising tool to develop DC-based vaccination strategies, there are several factors, which we will discuss below, that have so far hampered the applicability.

## Cross-Presentation of Immune Complexes and the Diversity of Fc Receptors

A major difficulty for studying and determining the therapeutic applicability of cross-presentation of immune complexes is the complexity of the Fcγ receptor family [Table 1; Ref. (29)]. Several types of Fc receptors have been found in addition to species-dependent differences. In mice, four different classes of Fcγ receptors comprising FcγRI, FcγRIIB, FcγRIII, and FcγRIV have been described. The activating Fc receptors FcγRI, FcγRIII, and FcγRIV consist of an immunoglobulin binding α-chain and a signal transducing γ-chain, which carries an immunoreceptor tyrosine-based activation motif (ITAM). In contrast, FcγRIIB is a single chain inhibitory receptor with an immunoreceptor tyrosine-based inhibitory motif (ITIM). The human FcγR system seems to be far more complex as exemplified by the presence of gene families for FcγRI and FcγRII, as well as the presence of several allelic forms for FcγRIIIA, FcγRIIIB, and FcγRIIB. Mouse FcγRIV is most closely related to human FcγRIIIA whereas mouse FcγRIII is most similar to human FcγRIIA. FcγRIIIB is unique for the human system, but both species have the inhibitory function of FcγRIIB in common.

**Table 1:** **Overview of human and murine Fcγ receptors**.

Human/mouse	IgG receptor	CD	Function	Affinity	Structure
Human (30–33)	FcγRIIA	CD32A	Activation	Low to medium	α-Chain with ITAM
	FcγRIIC	CD32C	Activation	Low to medium	α-Chain with ITAM
	FcγRIIIA	CD16A	Activation	Low to medium	α-Chain and γ_2_-chains with ITAM
	FcγRIIIB	CD16B	Activation	Low to medium	GPI-linked α-chain
Human and mouse (30–33)	FcγRI	CD64	Activation	High	α-Chain and γ_2_-chains with ITAM
	FcγRIIB	CD32B	Inhibition	Low to medium	α-Chain with ITIM
Mouse (30–33)	FcγRIII	CD16	Activation	Low to medium	α-Chain and γ_2_-chains with ITAM
	FcγRIV		Activation	Low to medium	α-Chain and γ_2_-chains with ITAM

Dendritic cells simultaneously express activating and inhibitory Fc receptors [reviewed in Ref. (18)]. The conserved expression of an inhibitory Fc receptor along with activating Fc receptors among species suggests that Fc receptor-mediated cross-presentation is tightly regulated *in vivo*. The requirement of strictly controlling Fc receptor-mediated cross-presentation was demonstrated by studies that show that antibody-mediated cross-presentation of self-antigens contributes to autoimmune disease (34, 35). The authors looked at the development of autoimmune diabetes in RIP-OVA mice. In this model, the transfer of OVA-specific naïve CD8^+^ T cells induces peripheral tolerance. Importantly, the co-administration of anti-OVA IgG leads to CD8^+^ T cell-driven diabetes through the activating Fcγ receptors on DCs. The disease pathogenesis in this model was further augmented in FcγRIIB knockout mice, suggesting a tolerogenic function of FcγRIIB *in vivo*. In line with a tolerogenic function of this receptor, it was shown that DCs from FcγRIIB knockout mice generate overall stronger immune responses and that blocking immune complex binding to FcγRIIB promotes DC maturation, which is considered one of the most important factors for efficient priming of CTL responses (36–39). This suggests that expression of inhibitory FcγRIIB, which restricts DC maturation under non-inflammatory conditions and thus probably prevents autoimmunity, may hamper immunotherapeutic approaches against tumors and microbial infections (29, 40). Hence, it is important to be aware of the expression patterns and ratios of activating versus inhibitory Fc receptors on murine and human DCs when studying the effects of immune complexes.

Additionally, IgG subclass composition of immune complexes has been shown to influence binding affinity resulting in different binding properties to individual Fc receptors (41). For example, immune complexes composed of human IgG1 bind with relatively high affinities to all Fc receptors, whereas IgG2 immune complexes seem to bind primarily to human FcγRIIA and FcγRIIIA (42). Furthermore, disparities in the binding affinities of immunoglobulin isotypes for specific Fcγ receptors exist between mice and humans. Thus, predictions of immune complex functions drawn from wild-type mouse models might be inadequate. A prominent example of the failure of previous studies in accurately recapitulating the specificity and diversity of Fcγ receptor interactions is the outcome of a clinical trial using a CD28-specific superagonistic antibody; this led to severe side effects including severe pain and extreme swelling, as well as one individual suffering from heart, liver, and kidney failure (43). To address this problem, an FcγR humanized mouse strain was recently generated through transgenic expression of the entire human FcγR family under the control of their human regulatory elements on a genetic background lacking all mouse FcγRs (44). The animals demonstrate normal lymphoid tissue development and generate normal immune responses. Thus, this mouse strain offers a greatly improved model to study immune complex-mediated cross-presentation, although it addresses only the species-specific differences regarding Fcγ receptors. Humans and mice also display differences in the expression patterns of Fc receptors for IgE and IgA, which might contribute to cross-presentation of immune-complexed antigen *in vivo* (45–48).

Increasing evidence suggests that allelic isoforms and polymorphisms of Fc receptors are shaping immune responses in humans. FcγRIIA (CD32A), the major phagocytic FcγR in humans, exhibits a polymorphism in the ligand-binding domain (49). Individuals homozygous for the R allelic form of CD32A (CD32AR allele) have been described as more susceptible to bacterial infections and autoimmune diseases compared to individuals homozygous for the H allelic form of CD32A (CD32AH) and CD32AR/H heterozygous individuals (50, 51). A binding study using two-dimensional affinity measurements also demonstrated that compared to CD32AH, CD32AR has significantly lower affinity toward IgG2, as well as to IgG1 and IgG3, suggesting that the lower binding of CD32AR to IgGs might be responsible for the lack of immune complex clearance, which leads to increased susceptibility to bacterial infections and autoimmune diseases (52). Genetic variations in Fc receptors have also been linked to cancer susceptibility (53–55). However, less efficient immune complex binding might also be reflected in less efficient antigen uptake and presentation via this receptor, and thus consequences for immune complexes cross-presentation should be expected. Of note, glycosylations in the IgG–Fc region can also affect Fc receptor-binding properties as discussed in detail in a recent review (56). How antigen cross-presentation of immune complexes and T cell priming is altered by differences in IgG subclass composition, IgG–Fc glycosylation, and Fc receptor polymorphisms is currently unknown, but is important to address. In conclusion, the complexity of interactions of IgG with the Fc receptor system in addition to concerns about species specificity presents a major hurdle that needs to be overcome for successful therapeutic applications.

## Cross-Presentation of Immune Complexes and the Diversity of DC Subpopulations

Whether it would be beneficial to target a specific DC subset that displays a superior capacity to cross-present antigen for therapeutic approaches is currently a field of extensive investigation (4, 57). We will first focus on what we know so far about the cross-presentation capacity of DC subsets in general and then discuss our current understanding of cross-presentation of immune complexes in regard to DC subsets. DCs are a heterogeneous cell population, and substantial effort was made to characterize different subsets in mice and identify their human counterparts [reviewed in Ref. (58–60)]. In principal, murine and human DCs can be divided into two major subsets, classical/conventional DCs (cDCs) and plasmacytoid DCs (pDCs). In mice, cDCs comprise CD8α^+^ and CD8α^−^ lineages, which have been found to differ in their ontogeny and display functional specializations. Since the expression of surface markers on human and murine DCs is not conserved, only recently has gene expression profiling allowed for the identification of human CD141^+^ DCs as functional equivalents of the mouse CD8α^+^ DCs, while human CD1c^+^ DCs appear to be comparable to mouse CD8^-^ DCs (61, 62).

In mice, the CD8α^+^ DC subset is considered to be more efficient at antigen cross-presentation than other DC subsets (63–66). The corresponding human subset, CD141^+^ DCs, is also potent at inducing CD8^+^ T cell responses *in vitro*, although their superiority to other human DC subsets is uncertain (67–73). Several groups have now reported that all human DC subsets can efficiently cross-present several forms of antigen [reviewed by Ref. (57)]. Initially, CD141^+^ DCs isolated from human blood were described to better cross-present CMV protein pp65 in comparison to CD1c^+^ DCs and pDCs from the same donor (67). It is important to note, however, that cross-presentation *in vivo* occurs rather in secondary lymphoid organs. A recent study has overcome the difficulties in isolating sufficient amounts of human DCs from lymphoid tissue and characterized in detail the cross-presentation properties of tonsil-resident DCs (73). An important finding of this study was that all tonsillar DC subsets (i.e., pDCs and the two populations of cDCs, CD1c^+^ DCs and CD141^+^ DCs) displayed comparable capacities to cross-present soluble antigens in contrast to macrophages, which lacked this ability. Interestingly, necrotic cells were phagocytosed and cross-presented by CD1c^+^ DCs and CD141^+^ DCs with similar efficiency, while pDCs were poor at taking up necrotic particles, consequently resulting in inefficient cross-presentation. Tonsillar macrophages were found to be the most efficient at taking up dead cells, but despite this fact they completely failed to cross-present necrotic cells. Collectively, the ability to efficiently cross-present in humans seems less restricted to a specific DC subpopulation than as observed in mice. Along these lines, it has been shown that the cross-presentation properties of human DCs depend on the antigen uptake pathway and the ability of the pathway to route the antigen into an early endosomal compartment rather than on a specific DC subset (74, 75). CD141^+^ DCs are superior cross-presenters compared to CD1c^+^ DCs only when the antigen is delivered via CD205, a receptor that preferentially targets antigens to late endo/lysosomal compartments. If antigen is targeted through CD40, CD1c^+^ DCs are as efficient as CD141^+^ DCs. These findings argue that targeting one specific DC subset for the design of DC-based vaccines may not offer the presumed advantage.

The cross-presentation studies discussed above focused primarily on soluble antigen uptake and targeting antigen via several endocytic receptors. How does cross-presentation of immune complexes fit into this picture? Targeting DCs through IgG immune complexes has been proven to be superior to soluble immune complexes for inducing CD8^+^ T cell responses and as anti-tumor vaccines by utilizing murine bone marrow-derived DCs (76, 77). In addition, circulating specific antibodies have been shown to enhance systemic cross-priming by delivering immune-complexed antigen to murine DCs *in vivo* (78). Notably in mice, immune-complexed antigen allows the CD8α^−^ DC subset, which has been proven to be very poor at presenting soluble antigen, to become potent cross-presenting cells (79). Interestingly, cross-presentation by CD8α^−^ DCs depends on activating Fcγ receptors. Lack of the signal transducing γ-chain specifically abolishes presentation of immune-complexed antigen on MHC class I molecules but not on MHC class II molecules (79). Another remarkable feature regarding cross-presentation of immune complexes is their reliance on FcRn, an IgG binding receptor that is primarily located intracellularly and binds IgG independently from their Fcγ receptor interaction sites (80). How FcRn promotes cross-presentation of immune complex is discussed later in more detail.

Our knowledge regarding cross-presentation of immune-complexed antigen by human DC subsets is still very limited. The effects of Fcγ receptor antigen targeting on the efficiency of cross-presentation in human DCs were recently investigated using human cytomegalovirus (HCMV) pp65 as a protein antigen (81). In line with the data obtained from murine models, immune-complexed antigen is more efficiently cross-presented than comparable amounts of soluble antigen by human DCs. The enhanced cross-presentation capacity observed was not mediated by increased antigen uptake or induction of DC maturation through the immune-complexed antigen. The authors also demonstrated that both of the two major intracellular cross-presentation pathways (4), the cytosolic and the vacuolar/endosomal pathway, are involved in Fcγ receptor-mediated uptake of immune complexes and their processing. Notably, monocyte-derived DCs as well as CD141^+^ DCs required antigen processing by both intracellular pathways. The finding that CD141^+^ DCs, which are the human equivalent to CD8α^+^ DCs, use both processing pathways for immune complexes points to unique features of human DCs. Murine CD8α^+^ DCs mainly use the cytosolic pathway to process antigen for cross-presentation, including the processing of immune complexes (82). Another difference to murine DCs is that the CD141^+^ DC subset proved to be superior to CD1c^+^ DCs in cross-presenting pp65 immune complexes (81). These findings point to obvious differences between murine and human DC subsets regarding immune complex-mediated cross-presentation. Since the human DCs were isolated from blood (81) and the murine DCs were isolated from the spleen (79, 80), it is possible that DCs from blood and lymphoid tissue generally differ in their cross-presentation capacities of immune complexes, which have similarly been observed for human DC subsets in response to soluble antigen as described above. In any case, the study by Flinsenberg et al. found that Fcγ receptor targeting increases cross-presentation of HCMV antigen by human blood and tonsillar CD141^+^ DCs, which suggest that targeting of this DC subset with immune complexes might improve DC-based vaccination strategies. Another very important aspect of this study is the detailed characterization of Fcγ receptor expression on human DC subsets. Although CD1c^+^ DCs expressed overall higher levels of FcγRII, CD141^+^ DCs seem to express higher levels of the activating FcγRIIA relative to the inhibitory FcγRIIB. Thus, this study clearly demonstrates that the overall expression level of one specific Fcγ receptor does not determine the functional outcome, and that we need to consider the diversity of Fcγ receptor expression by distinct DC subsets to evaluate the therapeutic potential of immune complex-mediated cross-presentation.

A further difference between mice and humans seems to be the cross-presentation capacity of pDCs. Several studies have reported that murine pDCs do not possess the ability to cross-present (83–86) or that their capacity is insignificant when compared to cDCs (87). In contrast to mouse pDCs, human pDCs can efficiently cross-present antigen and induce CD8^+^ T cell responses (88–90). Human pDCs also express FcγRIIA, and this receptor has been shown to mediate internalization of immunoglobulins bound to chromatin (91), Coxsackie virus (92), the model antigen KLH (93), and the tumor antigen NY-ESO-1 (94). In addition, the group of de Vries described that pDCs can use several receptor-targeted antigen uptake pathways, including the activating FcγRIIA receptor, to target antibody-coated nanoparticles for cross-presentation. Although this study did not use classical immune complexes, together with a vaccination study in which pDCs significantly prolonged overall survival in melanoma patients (95), it supports the notion that pDCs are interesting targets for DC-based immunotherapeutic strategies.

Collectively, we should keep in mind that some of the observed differences between human and murine DC subsets regarding cross-presentation of immune complexes most likely stem from differences in their Fc receptor expression and from different binding affinities for IgG isotypes. Recently, various published and publicly available microarray data were compiled, and this mRNA collection provides an excellent overview of mouse and human Fcγ receptor expression by DC subsets, monocytes, and macrophages (18). Overall, the Fcγ receptor expression levels obtained by mRNA analysis correspond well with the surface expression levels acquired by flow cytometric analysis (FACS) (Table 2). For the future, it will be important to determine whether the Fcγ receptor expression of human DC subsets isolated from blood also matches the expression on tissue-resident DCs from different organs.

**Table 2:** **Fcγ receptor expression by murine and human DC subsets**.

Human DCs				Mouse DCs			
	Expression				Expression		
	High: +++; low: +				High: +++; low: +		
DC subset	Receptor	FACS^a^	mRNA^b^	DC subset	Receptor	FACS (79, 80, 89)	mRNA^b^
CD141^+^ (BDCA3^+^, XCR1^+^)	FcγRI	−	−/+	CD8^+^	FcγRI	−/+	+
	FcγRIIA	+	−/+		na		
	FcγRIIB	+	+		FcγRIIB	+++	++
	FcγRIIIA	−	+		FcγRIII	+++	+
	na				FcγRIV	−/+	+
CD1c^+^ (BDCA1^+^, SIRPα^+^)	FcγRI	+^c^	+	CD8^−^	FcγRI	−/+	+
	FcγRIIA	++	+++		na		
	FcγRIIB	+++	+++		FcγRIIB	++	++
	FcγRIIIA	−/+	+		FcγRIII	++	+
	na				FcγRIV	−/+	+
pDCs	FcγRI	−	−/+	pDCs	FcγRI	−	+
	FcγRIIA	++	+		na		
	FcγRIIB	+	+		FcγRIIB	+	++
	FcγRIIIA	nd	+		FcγRIII	−	+
	na				FcγRIV	−	+
Monocyte-derived DCs	FcγRI	+	+	Bone marrow-derived DCs	FcγRI	−/+	++
	FcγRIIA	++	+++		na		
	FcγRIIB	+++	+++		FcγRIIB	++	−/+
	FcγRIIIA	−/+	+		FcγRIII	++	++
	na				FcγRIV	−/+	++
Slan DCs (CD16^+^)	FcγRI	++	nd	na			
	FcγRIIA	++					
	FcγRIIB	+					
	FcγRIIIA	+++					

*^a^Published surface expression determined by flow cytometric analysis (FACS) (81, 96–98)*.

*^b^mRNA data from compiled microarrays (18)*.

*^c^CD1c^+^ DCs isolated from blood; tonsillar CD1c^+^: DC −/+*.

*nd: not determined*.

*na: not applicable*.

## Regulation of Fcγ Receptor Expression Impacts Cross-Presentation of Immune Complexes

Efficient cross-presentation for inducing protective immune responses against tumors or viruses is strongly governed by the ratio of activating versus inhibitory Fcγ receptors expressed on DCs. In addition to the DC subset, the maturation/activation state of DCs likely impacts their Fcγ receptors expression pattern. The maturation/activation state of DCs is in general strongly influenced by the cytokine milieu of the microenvironment, and a considerable number of cytokines have been shown to regulate Fcγ receptor expression *in vitro*. TGF-β1 down-regulates surface expression FcγRI and FcγRIII on monocytes (99). IL-4, a cytokine associated with Th2-type immune responses, increases the expression of inhibitory FcγRIIB. In contrast, the Th1-cytokine IFN-γ increases expression of activating Fc receptors on monocytes (100). Monocytes also have been shown to respond to IFN-γ and TNF-α treatment with enhanced immune complex binding via FcγRI, even when saturated with pre-bound monomeric IgG (101). Cytokine-induced changes in Fcγ receptor expression were also found using monocyte-derived DCs (96). Immature DCs generated with GM-CSF and IL-4 from monocytes express high amounts of inhibitory FcγRIIB, which is down-regulated upon DC maturation induced by TNF-α. The authors also showed that blood DCs activated with a cytokine cocktail containing TNF-α, IL-1β, IL-6, and PGE2 induce more influenza-specific CD8^+^ T memory cells via targeting of FcγRI and FcγRIIA. Interestingly, crosslinking of inhibitory FcγRIIB only reduced the cross-presentation ability of immature DCs but not of mature DCs. Treatment of mature blood DCs with IL-10, or a combination of IL-10 and IL13, was found to increase expression of FcγRIIA and FcγRIIB (96). To sum up, although we know that cytokines can modulate Fcγ receptor expression, and that tumors create cytokine-rich microenvironments that involve the production of immunosuppressive as well as inflammatory cytokines to drive tumor progression (102, 103), our knowledge is very limited as to how cytokines from the tumor microenvironment affect cross-presentation of immune complexes by DCs. Thus, regarding anti-tumor therapy, this gap in knowledge might explain why the long-term therapeutic outcomes of immune complex-based strategies were not more successful, although efficient cross-presentation is induced by IgG-complexed antigens. One explanation could be that the tumor microenvironment promotes the induction of cross-tolerance by keeping the DCs in an immature state, which is associated with high expression levels of inhibitory FcγRIIB. Another possible scenario would be that immune complex-mediated cross-presentation via activating Fcγ receptors, which is known to result in inflammatory cytokine production by the DCs, actually contributes to an inflammatory tumor microenvironment, which fosters tumor progression by supporting, for example, angiogenesis. Therefore, future studies are needed that not only address which activating and inhibitory Fcγ receptors are expressed by DC subsets, but also define how their expression patterns are regulated and which cytokines are induced by DC subsets after immune complex-mediated activation *in vivo*.

## FcRn – An Intracellular Relay Receptor That Guides Cross-Presentation of IgG-Containing Immune Complexes

In general, little is known about the intracellular mechanisms that are involved in processing of immune-complexed antigen for cross-presentation. Substantial evidence exists for an important role of FcRn in the cross-presentation of IgG-containing immune complexes. FcRn, which is an MHC class I-like molecule, was initially described only in intestinal epithelial cells of neonatal rodents, but it has since been shown to be expressed throughout life in several cell types, including human and rodent DCs (104–106). If CD8α^−^ DCs do not express FcRn because of genetic alterations, the cell loses its ability to efficiently cross-present and fails to elicit CD8^+^ T cell responses (80). Elegant studies showed that FcRn regulates the intracellular sorting of IgG immune complexes in CD8α^−^ DCs. In contrast to CD8α^+^ DCs where the endosomes are buffered around the neutral pH of 7.0 that prevents antigen degradation and promotes cross-presentation, Fcγ receptors in CD8α^−^ DCs traffic antigens into acidic compartments (pH 6.0). The acidic environment is, by itself, not favorable for cross-presentation; however, it favors the binding of IgG to FcRn, and thus the model proposes that FcRn traps immune-complexed antigen and protects it from degradation within an acidic loading compartment. The study also showed that in parallel to antigen entry into the FcRn-positive compartment, key components of the phagosome-to-cytosol cross-presentation machinery are rapidly recruited to the endo/lysosome. Vesicles that contained IgG-opsonized particles or IgG immune complexes rapidly acquired greater quantities of vacuolar ATPase (V-ATPase), gp91phox, and Rab27a than those that resulted from internalization of IgG mutants that cannot interact with FcRn. Consistent with this concept, it was described that the presence of FcRn also affects the oxidation state as well as the acidification of vesicles. Inhibitor studies demonstrated that FcRn-mediated cross-presentation depends on the proteasome as well as Sec61α, which is indicative for the cytosolic cross-presentation pathway. Since insulin-regulated amino peptidase (IRAP) enrichment was not depicted in FcRn-positive IgG immune complex-containing vesicles, and cathepsin inhibitors did not abrogate IgG immune complex cross-presentation, the authors concluded that the alternative vacuolar pathway was not involved. In summary, this study suggests that FcRn binding of IgG immune complexes enables a slower and more controlled antigenic degradation in CD8α^−^ DCs, thereby permitting this population of DCs to become efficient cross-presenting cells.

The most compelling evidence for the exceptional importance of FcRn for cross-presentation of IgG immune complexes and IgG-opsonized particles is derived from *in vivo* studies that analyzed the effects of FcRn-deficiency on chronic intestinal inflammation and colonic cancer (107, 108). In a chemically induced chronic colitis model, which is associated with generating high levels of anti-bacterial antibodies that enter the host as IgG immune complexes, Baker et al. demonstrated that FcRn-dependent cross-presentation is carried out by CD8α^−^ DCs *in vivo*, leading to greater levels of cytotoxic T cell activation during the course of colitis. In a recent study, the same group focused on the impact of FcRn on tumor development, clearly demonstrating the importance of this molecule for anti-tumor immune surveillance (108). The authors found that the DC-specific deletion of FcRn leads to increased tumor burden in experimental models of colon cancer and lung metastasis. Strikingly, this study also demonstrated that colon cancer patients with higher numbers of FcRn-positive DCs in the adjacent tumor tissue had significantly better prognoses, confirming the crucial role of FcRn and demonstrating the vital role of cross-presentation of IgG immune complexes in anti-tumor immunity in general. It will now be of utmost importance to elucidate the details of the intracellular mechanism of this process to evaluate whether the pathway can be employed for cancer immunotherapy.

## Conclusion

Although ample evidence suggests that Fcγ receptor targeting through immune complexes allows for more efficient cross-presentation compared to soluble antigen, it still needs to be proven which advantages it may have over targeting of other endocytic receptors on DCs, especially *in vivo*. In this respect, it is very important to continue developing better murine models which more accurately reflect the human immune system. The recently published humanized FcγR mouse strain is here a promising step in the right direction. For therapeutic manipulations, we also need to better understand how Fcγ receptor expression by DCs is regulated. Can we use cytokines and/or TLR ligands to modulate the ratio of inhibitory versus activating Fcγ receptors expressed by DC subsets to improve therapeutic strategies? TLR-2 ligands, for example, have been shown to increase expression of inhibitory FcγRIIB in macrophages (109), a consequence not desirable in the context of viral or tumor vaccine development. Furthermore, how does the size of immune complexes influence cross-presentation? How does the antibody to antigen ratio in immune complexes influence cross-presentation? Indeed, it has been shown that immune complex size and glycosylation on IgG impact the binding to human Fcγ receptors (110). In summary, it is fair to conclude that many important questions remain open and need to be addressed. Irrespectively, cross-presentation of immune complexes represents an exciting potential pathway to improve DC-based vaccination strategies for anti-viral as well as anti-tumor therapy.

## Conflict of Interest Statement

The authors declare that the research was conducted in the absence of any commercial or financial relationships that could be construed as a potential conflict of interest.
